# Comparison of the Fatty Acid Profiles of Sow and Goat Colostrum

**DOI:** 10.3390/vetsci11080341

**Published:** 2024-07-29

**Authors:** Lucía Ayala, Pilar Gómez-Cortés, Fuensanta Hernández, Josefa Madrid, Silvia Martínez-Miró, Miguel Angel de la Fuente

**Affiliations:** 1 Department of Animal Production, Faculty of Veterinary, International Excellence Campus for Higher Education and Research “Campus Mare Nostrum”, University of Murcia, 30100 Murcia, Spain; lucia.ayalag@um.es (L.A.); nutri@um.es (F.H.); alimen@um.es (J.M.); silviamm@um.es (S.M.-M.); 2 Department of Bioactivity and Food Analysis, Institute of Food Science Research (CIAL, CSIC-UAM, CEI UAM+CSIC), 28049 Madrid, Spain; mafl@if.csic.es

**Keywords:** energy supplement, goat colostrum, short-chain fatty acids, medium-chain fatty acids, lipid composition

## Abstract

**Simple Summary:**

In intensive dairy goat farms, artificial lactation is used for kids, often with only the colostrum from the first milking being used, discarding the milk from the second milking, or transition milk. On the other hand, in modern pig breeds, the number of piglets born with low birth weight is increasing, mainly due to the growth in litter sizes. These piglets are less likely to survive in the first hours of life due to their low energy reserves, among other factors. The aim of this study was to improve the current knowledge of goat colostrum for its use as an alternative energy supplement for newborn piglets. In order to assess the similarity of sow and goat colostrum, the fatty acid (FA) composition of both was examined in detail. The major FAs (palmitic, oleic, and linoleic acids) were the same in both species, although they were found in different proportions. Goat colostrum is mainly characterized by a high proportion of short- and medium-chain FAs (4:0, 6:0, 8:0, and 10:0), which are absent in sow colostrum. This group of FAs may play an important role in colostrum, as they are a readily available source of energy and have also been attributed to strong antibacterial activity. Therefore, the surplus of goat colostrum and milk from the second milking not used for feeding kids could be a promising energy supplement for newborn piglets, especially for the weakest and smallest of the litter, which are the most in need.

**Abstract:**

Currently, the utilization of hyperprolific sows has stimulated the search for supplements aimed at enhancing piglet survival, as these sows yield more offspring than they can adequately feed with their colostrum production. In contrast, intensive goat farming often yields surplus colostrum, thus necessitating its removal, since kids are exclusively fed colostrum through lactation solely within the initial day of birth. The objective of this study was to examine and compare the fatty acid (FA) profiles of colostrum from sows and goats, together with possible influencing factors such as sow parity and the postpartum day of the goat, for possible use as an energy supplement for neonatal piglets. Swine colostrum was collected from sows with a 0–5 parity. In addition, samples of goat colostrum were collected on their first (D1) and second (D2) days of postpartum milking. The FA profiles of the colostrum were analyzed via gas chromatography. The parity value of the sows did not affect (*p* > 0.05) the FA colostrum composition. High proportions of palmitic, oleic, and linoleic acids were found in both types of colostrum. Levels of palmitic, oleic, and linoleic acids were significantly higher in D1 goat colostrum, whereas saturated FAs of less than 14 carbons (4:0, 6:0, 8:0, 10:0, and 12:0) were found in higher proportions in D2. These FAs play an important role in colostrum as they are a readily available source of energy and have also been attributed strong antibacterial activity. Therefore, goat colostrum, especially D2, could be used as an alternative energy supplement for newborn piglets, in particular for the weakest and smallest of the litter, which are the most in need.

## 1. Introduction

Colostrum is a rich source of nutrients and bioactive compounds that play a key role in thermoregulation, intestinal development, and immunity acquisition in piglets. Due to the increase in litter size in modern pig breeds, the number of piglets born at a low birth weight, typically under 1.0 kg, is increasing [1,2]. Such low birth weight leads to high rates of pre-weaning morbidity and mortality, as well as permanent delays in growth and development [3,4]. These piglets have a lower chance of survival because of their lower body energy reserves, among other factors. Unfortunately, colostrum yield is not affected by litter size and, consequently, is distributed among a higher number of piglets in modern hyperprolific sows, leading to insufficient intake, especially by less competitive and lower weight animals [5,6].

Colostrum provides highly metabolizable energy to newborn piglets, which is used to combat hypothermia, very common in the first hours of life. Colostrum intake increases the metabolic rate and maintains the homeothermic balance in piglets during the first day after birth, and is also beneficial to their development and growth [7,8,9]. The most important energy component in colostrum is lipids, which account for 5–8% in the first secretions and change significantly over time [10]. Triacylgycerols (TGs) are the main lipids in sow milk, accounting for 94–96% [11], and have the primary function of providing energy. In addition, TGs present essential fatty acids (FAs) crucial for maintaining the normal function of the body. The FA content and composition of sow colostrum are influenced by different factors, including genetics, lactation status, and maternal nutrition [12,13].

Since pre-weaning mortalities occur mainly in the first two days postpartum, nutritional supplementation at birth is one of the prioritized targets of recent research in the pig sector [14,15]. To deal with this problem, several nutritional supplements have been used: sow colostrum and its derivatives [16], cow’s milk and colostrum [17], and energy supplements based on vegetable oils [1] or glucose [18]. However, goat colostrum has barely been studied as a supplement for newborn piglets. Its higher digestibility could lead to a higher absorption and efficient utilization of nutrients. Furthermore, goats produce colostrum for much longer (36 h) and in greater quantity than sows [19]. Kids, unlike piglets, are separated from goats as soon as they are born, and they receive colostrum administered mechanically, without even using 100% of the colostrum produced on the first day postpartum. Therefore, intensive dairy goat industries produce thousands of liters of colostrum that is normally discarded, which is an important economic and environmental problem.

The lipid composition of milk from both ruminant and non-ruminant mammals has been widely studied, as has the profile of major and essential FAs in colostrum. However, a complete FAs profile, particularly in monogastric animals such as sows, has received scarce attention [20]. Since the sow and the goat are different species, it is important to conduct a detailed comparative study of the FA composition of both types of colostrum beforehand. The aim of this study was to determine the complete FA profiles in goats and sows, as well as certain factors that could affect them, such as the parity of the sow and the postpartum day of the goat. Through this method, we aimed to improve our understanding of goat colostrum and establish the hypothesis about its potential as a possible alternative energy supplement for newborn piglets.

## 2. Materials and Methods

### 2.1. Colostrum Collection

The animal study protocol was approved by the Ethical Committee of Animal Experimentation of the University of Murcia, Spain (904/2023), in accordance with the Royal Decree 53/2013 of 1 February 2013, which established the basic rules applicable to the protection of animals used for experimental and other scientific purposes, including teaching.

Colostrum samples were obtained from two commercial farms located in the southeast of Spain. Colostrum sampling was conducted based on the standard management practices for each species: manual milking for pigs and mechanical milking for goats. Hormones were not used to collect sow or goat colostrum.

Individual porcine colostrum samples (50 mL per sow) were collected from sows from the same farrowing batch. A total of fifteen sows (Landrace × Large White) of different parities (5 first-farrowing sows, 5 second-to-fourth farrowing sows, and 5 sows of fifth and more farrowing) were sampled. Colostrum samples were taken when the first piglet was expelled. Before colostrum sampling, the entire visible udder was cleaned with soap and water and dried with paper. Subsequently, colostrum was collected through manual milking by massaging evenly [10]. During gestation, sows were fed with 2.5 kg/sow of a commercial gestation diet offered in meal, containing 12.2 MJ/kg of metabolizable energy (ME), 130 g/kg of crude protein, and 6 g/kg of lysine [21] and without ingredients of animal origin.

Bulk goat colostrum samples (100 mL) were collected in batches of fifty multiparous Murciano-Granadina goats via mechanical milking. Colostrum samples were taken from the first and second milking, corresponding to the first (D1) and second (D2) postpartum days. Five different goat batches were sampled to obtain five replicate colostrum bulk samples. Goats were fed during pregnancy with a forage (based on corn silage)-to-concentrate ratio of 53:47. Daily intake was 1.27 kg dry matter per animal, corresponding to 1.25 forage units for milk production (UFL, 1 UFL = 7.1128 MJ of net energy for lactation) [22]. All colostrum samples (sow and goat) were placed in sterile containers and immediately stored at 4 °C.

### 2.2. Chemical Composition Analyses and Immunoglobulin Quantification

The chemical composition of the colostrum samples was analyzed via infrared spectroscopy using a MilkoScan FT6000 Analyzer (Foss Electric, Hillerød, Denmark). Quantification of the immunoglobulins in the sow and goat colostrum was determined using commercial pig- and goat-specific IgG indirect enzyme-linked immunosorbent assay (ELISA) kits (Bethyl Laboratories, Inc., Montgomery, TX, USA; kit nº E101–104, E50–104). The assay showed intra-assay coefficients of variation below 15%.

### 2.3. Sample Processing

For each sample, aliquots of 10 mL were prepared. The aliquots were ultracentrifuged at 17,800× *g* for 30 min at 4 °C [23]. Fat was scraped into an Eppendorf tube with a metal spoon and stored at −80 °C until the FA profile was studied.

### 2.4. Fatty Acid Analyses

Fatty acid methyl esters (FAMEs) were prepared via base-catalyzed methanolysis of glycerides with KOH in methanol [24]. Gas chromatography was used to provide a complete FAME profile. An Agilent model 6890 N Network Gas Chromatograph (Palo Alto, CA, USA) equipped with auto-injector, flame ionization detector, and a CP-Sil 88-fused silica capillary column (100 m × 0.25 mm i.d., Varian, Middelburg, The Netherlands) was used. Injector and detector temperature was 250 °C. Helium was the carrier gas, and the split ratio was 1:100. Initial oven temperature was 45 °C. After 4 min, the oven temperature was raised from 13 °C min^−1^ to 165 °C, then a 35 min hold, then increased to 215 °C at 4 °C min^−1^, and finally maintained for 30 min. A quantification of individual FAMEs was performed using milk fat with known composition (CRM 164; European Community Bureau of Reference, Brussels, Belgium). Individual FAs were identified by conducting comparisons with standards distributed by Nu-Chek (Elysian, MN, USA) or based on chromatograms obtained under similar analytical conditions that were previously reported [25,26].

### 2.5. Statistical Analyses

SPSS Statistics 15.0 software (IBM SPSS, Chicago, IL, USA) was used for the statistical analyses. A one-way ANOVA analysis was used to determine the effect of parity on the FA profile of sow colostrum. Mean differences were evaluated using Tukey’s test. FA mean differences between D1 and D2 goat colostrum were compared using Student’s *t*-test. In addition, to compare the FA contents between the goat (D1 and D2) and sow colostrum, a heatmap was generated using the package “gplots” of the R software (v3.1.1). For all analyses, *p* < 0.05 was determined to be statistically significant.

## 3. Results

### 3.1. Chemical Composition of Colostrum

The chemical composition of the colostrum samples is shown in Table 1. A higher protein concentration and a higher IgG content were observed in sow colostrum compared to goat colostrum (*p* < 0.05). In contrast, a higher fat content was found in goat colostrum (*p* < 0.05).

### 3.2. Fatty Acid Profile of Sow Colostrum

Table 2 shows the average content of the diverse FAs in sow colostrum (expressed as % of total FAME) related to the effect of parity order. In general, no significant differences in colostrum composition were observed between sows of different parities (*p* > 0.05) with respect to saturated (SFA), monounsaturated (MUFA), and polyunsaturated (PUFA) groups.

More than 50 individual FAs were detected. Unsaturated fatty acids (UFAs) represented 68% of the total FAs, and SFAs constituted about 31%. Among UFAs, MUFAs constituted the most representative class (48% of total FA), whereas PUFAs were less abundant (21%).

Palmitic acid (16:0) was the most representative (22.74%) SFA, followed by stearic (18:0; 5.62%) and myristic (14:0; 1.48%) acids. Low amounts of odd-chain FAs such as 15:0 (0.14%) and 17:0 (0.33%) were also detected; and other even-chain FAs with more than 18 carbon atoms as 20:0, 22:0, and 24:0 were also identified below 0.15%. FAs with less than 10 carbon atoms were not found in sow colostrum. The branched SFA group represented up to 0.46% of the total FAs, and only iso 18:0 exceeded 0.1%.

With respect to MUFAs, oleic acid (*cis*-9 18:1) was the most abundant (36%), while other MUFAs such as *cis*-7 16:1, *cis*-9 16:1, and *cis*-11 18:1 were generally found in percentages below 5% (1.57, 3.67, and 4.94%, respectively). Other minor MUFAs, 14:1, 17:1, 20:1, and 22:1, all with *cis* geometric configuration, did not exceed 0.5%. Although small percentages of *trans* MUFAs were detected, their total contents were below 0.6% (Table 2).

Regarding PUFAs, linoleic acid (*cis*-9, *cis*-12; 18:2) was the most important quantitatively (16.27%). Low amounts of conjugated and non-conjugated 18:2 isomers were also found. None of these isomers had contents that exceeded 0.1%. Other omega-6 FAs found were 20:2 (0.39%) and γ-linolenic acid (18:3 n-6, 0.31%). Proportionally, omega-3 FAs were detected in lower amounts. The α-linolenic acid was the most abundant (0.91%), followed by 22:5 (0.36%), in percentages always below 1%. Omega-6/omega-3 ratios in all sow samples were high, with values greater than 11.

### 3.3. Fatty Acid Profile of Goat Colostrum

Table 3 shows the average SFA content of D1 and D2 goat colostrum. A total of 28 different SFAs were identified, representing about 68% of total FAs, of which 66.4% were non-branched SFAs and 1.3% were branched SFAs. Among the non-branched SFAs, palmitic acid (16:0) was the most abundant (30.34%), followed by 14:0 (11.66%), 18:0 (7.59%), 10:0 (5.98%), and 12:0 (2.96%). Other SFAs found were 4:0 (2.33%), 6:0 (2.08%), and 8:0 (2.02%). SFAs with more than 20 carbon atoms were found in amounts below 0.2%. Regarding branched SFAs, nine different FAs were identified, all in very low amount (<0.5%). There was an effect of the collection day on the SFA profile of goat colostrum: The total amount of SFAs was higher in D2 colostrum compared to D1, and the amount of short-chain SFAs up to 12 carbon atoms was also higher in D2 colostrum (*p* < 0.05). In contrast, 16:0 was higher in D1 when compared to D2 (*p* < 0.05).

Table 4 shows the average UFA content of D1 and D2 goat colostrum samples. MUFAs and PUFAs accounted about 28% and 4%, respectively. Regarding MUFAs, *cis* configuration accounted for 25% of total FAs, with *cis*-9 18:1 being the most abundant (21%), followed by *cis*-9 16:1 (1%) and *cis*-11 18:1 (1%). The other 13 *cis* MUFAs were detected at very low levels. Vaccenic acid (*trans*-11 18:1) was the most abundant *trans* MUFA (1.29%), followed by *trans*-10 18:1 (0.32%) and *trans*-12 18:1 (0.30%). The remaining *trans* MUFAs were found in smaller amounts (below 0.3%).

Concerning PUFAs, linoleic acid was measured in concentrations between 2.2 and 2.5%, while other non-conjugated as well as conjugated 18:2 FAs were detected in very low proportions. Rumenic acid (RA, *cis*-9, *trans*-11 18:2) was the most relevant conjugated FA found (0.5%). Concerning the remaining PUFAs, total omega-3 FAs accounted for 0.29% and total omega-6 for 2.94%, resulting in an omega-6/omega-3 ratio of around 10. 

The collection day affected the proportion of some FAs. Total MUFAs and PUFAs were higher in D1 colostrum (*p* < 0.05). Among the quantitatively major UFAs, there was also a statistically significant reduction in oleic and linoleic acids on D2.

### 3.4. Comparison of Swine and Goat Colostrum

**Figure 1:**
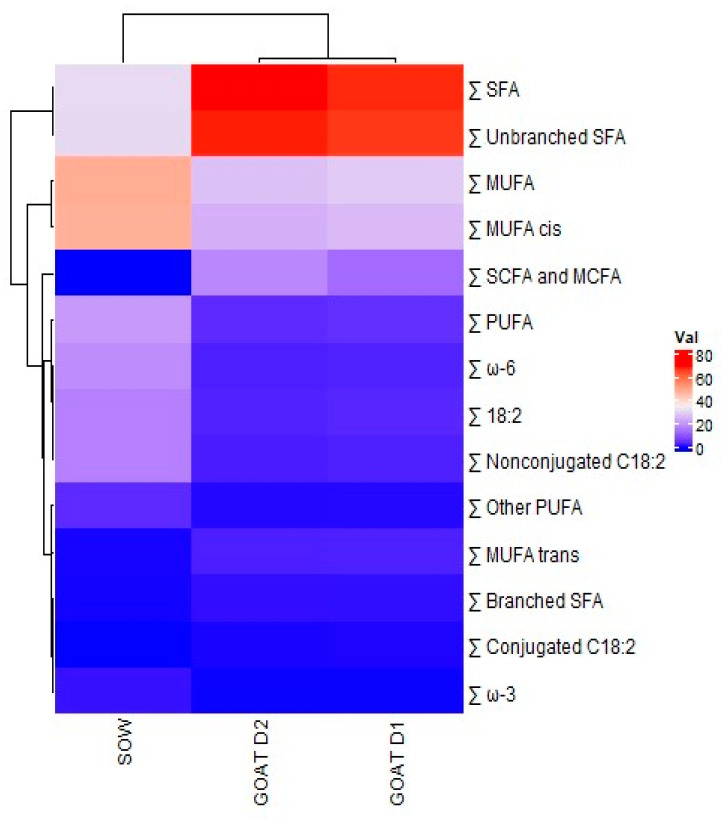
Heatmap of the fatty acid composition of sow and goat colostrum collected on the first (D1) and second (D2) days of postpartum milking. The color of each tile is proportional to the normalized detected concentration; red indicates an increased value, whereas blue indicates a decreased value. Clustering was performed by calculating the Euclidean distance.

The profile of the main FA groups of sow and goat colostrum (D1 and D2) is compared in the heatmap of Figure 1. The sum of SFAs, both branched and unbranched, was lower in sow colostrum (SOW) than in goat colostrum (D1 and D2) (*p* < 0.05), with no differences between D1 and D2 (*p* > 0.05). Short (SCFAs)- and medium (MCFAs)-chain SFAs were lower in sow colostrum when compared to goat (D1 and D2) (*p* < 0.05), but D1 was lower than D2 (*p* < 0.05). In contrast, sow colostrum was richer in PUFAs and MUFAs than goat colostrum (*p* < 0.05) as well as in total 18:2 and non-conjugated 18:2 (*p* < 0.05).

## 4. Discussion

This study aimed to deepen the knowledge of FAs in sow and goat colostrum. In dairy species, such as cow or goat, the FA profile has been studied more extensively [19,27,28]. In swine, the main components of colostrum have been studied, but detailed evaluations on the fat fraction remain scarce. In the present research, the FA profile of sow colostrum is scrutinized, and to our knowledge, this is among the most detailed studies in the literature on the matter [20].

The current work was conducted considering the number of farrowings of the sows, but no effect was observed on any of the FA main groups studied. These results contrast with the very few previously published studies on this subject. Luise et al. [11] detected significant differences in MUFA, PUFA, and SFA contents between colostrum samples from second- and fourth-farrowing Large White sows. Such differences were attributed to the different body conditions and energy balance between the second-parity-order sows and the more mature sows. 

Leaving aside parity effects, when considering the most relevant individual FA, the results obtained were similar to those previously reported [20,29]. The major SFA, and the second most abundant in sow colostrum, was 16:0. Palmitic acid in sow’s milk originally comes from blood plasma via body fat mobilization, although it can also be synthesized by mammary epithelial cells. Its intake by the newborn is of great importance as it is mainly involved in energy production and in replenishing body weight loss during periods of negative energy balance. It is worth mentioning that 16:0 is frequently present at higher levels in milk than in colostrum, independent of breed or diet [29,30,31]. This increase in 16:0 was found to contribute to the synthesis of the milk fat, which provided fractional energy for the growth of piglets [32]. On the other hand, small amounts of branched SFAs were detected, which was described for the first time in pig colostrum.

Oleic acid was the most abundant acid of the MUFA group, accounting for more than one-third of total FAs, similar to that described by other researchers [29,31,33]. The relevance of oleic acid lies in its oxidative capacity in the liver in the presence of glycogen, which is higher than that of SFAs such as 16:0 and 18:0 and which is decisive for the growth and thermoregulation of piglets [34]. The presence of *trans* MUFAs is not common in monogastric animals and has only been previously reported in sow colostrum by Luise et al. [11].

The PUFA contents quantified in our study in sow colostrum are in the range provided in previous research [20,29]. In addition to being a good source of energy, PUFAs have important structural functions as part of the phospholipid bilayer in cell membranes, affecting fluidity and intracellular signal transduction mechanisms [20]. They influence important physiological processes, including lipid mediator production, cell division, immune function, inflammation, and cell differentiation. Notably, sow colostrum contains higher proportions of PUFAs than transition and mature milk [31], supporting their importance for the early postnatal development of the piglet.

Previous research confirms the primacy of essential linoleic acid as the most important PUFA in sow colostrum. However, the percentages reported in the literature for this FA as well as for other PUFAs present variable ranges [20,29], which are attributable to the different sow diets [12]. Due to the ability of monogastrics to directly utilize dietary FAs for body fat synthesis, the composition of porcine colostrum is affected by the FA composition of the sow’s diet. Despite this fact, the PUFA content in sow colostrum is higher in comparison to goat colostrum, which presents a proportion close to 4%, caused by the ruminal digestion of TGs. This percentage is adequate for the proper development of the goat kids [27].

Regarding goat colostrum, the wide range of FAs identified is not surprising, since the milk fat of ruminants differs from that of other mammals, containing more than 400 different molecules, a diversity attributable to both the lipid metabolism of rumen microorganisms as well as the internal enzyme activity of the mammary gland [35]. 

Lactation time has been described as having an effect on the FA composition of goat milk. Marounek et al. [27] already observed an increase of 4:0, 6:0, 8:0, 10:0, and 12:0, together with a decrease of 16:0, in goat colostrum between days 1 and 2 of lactation. An identical trend has also been reported more recently between days 1 and 4 of lactation for both 16:0 and FAs from 4:0 to 12:0 [36]. This behavior has been attributed to the different degrees of expression of the enzymes involved in the FA synthesis in the mammary gland.

The presence of *trans* MUFAs, mainly vaccenic acid, is very rare in monogastric animals, but common in ruminants. Its origin is closely related to the ruminal biohydrogenation of dietary PUFAs. Through the desaturation of vaccenic acid, relevant amounts (around 0.5%) of rumenic acid, generated mostly in the mammary gland and with potential health benefits, were found [37]. The low proportion of other PUFAs, including omega-3 FAs, is already well known in ruminants, as goats [28].

The FA profile shown in goat colostrum may represent a nutritional advantage for neonatal piglets. Because colostrum TGs containing short- and medium-chain SFAs can be absorbed intact into intestinal epithelial enterocytes and then hydrolyzed by microsomal lipases, they would be a readily available source of energy, capable of improving the intestinal mucosal structure [38]. In particular, butyric acid is a primary energy source for intestinal epithelial cells with an essential role in the maintenance of colonic homeostasis and health [37]. Furthermore, short- and medium-chain SFAs are also characterized by strong antibacterial activity due to their ability to penetrate the semi-permeable membranes of bacteria and damage their internal structures.

The branched-chain SFA content of goat colostrum—three times higher than that observed in sow colostrum—could also represent an advantage for piglets from a physiological point of view. Although there are limited data on the specific effects of branched-chain SFAs in piglets, their positive role as health-protective bioactive components in humans is well known (the piglet is often chosen as an infant human model of the gastrointestinal tract because their physiology and metabolism are similar) [39]. Branched-chain SFAs are essential molecules in the digestive tract at the final stages of fetal development and after delivery. Apart from their function as antibacterial agents against certain pathogens, branched-chain SFAs may also play an important role in the growth and metabolism of enterocytes. They can be incorporated into the membrane of these cells, conferring them an anti-inflammatory activity [39].

Therefore, it is likely that goat colostrum can be a good supplement for newborn piglets due to its richness in short-/medium-chain as well as branched-chain SFAs. Short- and medium-chain SFAs are present in greater quantities in D2 colostrum compared to D1. D2 colostrum could preferentially be used as an energy supplement for piglets, since it is the colostrum that is currently discarded on goat farms. Although the use of goat colostrum in feeding piglets yet must be proven in actual feeding studies, considering all of this, D2 colostrum holds great promise as an effective energy supplement to reduce piglet mortality in the first 72 h of life.

Regarding UFAs, MUFAs were the most abundant in the colostrum of both species, and although in different proportions, *cis*-9 18:1 clearly stood out. The most abundant PUFA was linoleic acid and its content in sow colostrum was more than five times higher in comparison to goats. The remaining individual PUFAs were detected in percentages of no more than 1% in both species. Goat colostrum would not be sufficient to provide substantial amounts of omega-3 FAs (mainly α-linolenic, EPAs, and DHAs) to neonatal piglets, although it would contribute to a slightly lower omega-6/omega-3 ratio in the diet, which would be positive from a nutritional point of view [31]. In addition, goat colostrum would provide small amounts of *cis*-9 *trans*-11 18:2, with potential nutritional advantages for the newborn [37].

From the above, it can be seen that the FA profile of goat colostrum could be nutritionally beneficial for neonatal piglets. In addition, the fat globules are smaller in goat milk or colostrum than in that of cows, which makes them easily digestible because of their higher surface area [40]. In both species, the fat globules range from 1 to 10 µm, but the amount of fat globules smaller than 5 µm constitutes 60% in cow milk, whereas it is about 80% in goat milk [28]. Apart from their diameter, the smaller fat globules of goat milk are better dispersed, naturally homogenized, and provide a greater surface area of fat for rapid digestion by lipases [28].

Although there have been very few studies on this subject, the research conducted by Martínez-Miró et al. [41] showed that newborn piglets tolerated goat colostrum well, had no instances of diarrhea, and displayed no signs of intolerance when this colostrum was given during the first hours of life. Furthermore, these authors also demonstrated that neonatal piglets are capable of absorbing IgG from goat colostrum during the first hours after birth [41]. The correct absorption of immunoglobulins is a crucial factor in mammals, especially in species like goats, cattle, and pigs, where the nature of their placental structure necessitates the essential role of immunoglobulins in transferring passive immunity from mother to offspring. Numerous studies have demonstrated that immunoglobulins from colostrum, particularly bovine, are absorbed intact by other species [42]. Therefore, colostrum can be utilized therapeutically in both veterinary and human medicine, providing protection against diseases across a wide range of species [43]. Thus, all this evidence would support goat colostrum as a promising alternative to be used as new feed supplements or in the artificial rearing of newborn piglets.

## 5. Conclusions

Sow and goat colostrum have different FA profiles, although this does not make them incompatible from a nutritional point of view. The FA composition of goat colostrum could be suitable for supplementing the feed of newborn piglets and thus contribute to their survival during the first hours of life. The significant amounts of short- and medium-chain SFAs as well as other minor FAs present in goat colostrum could contribute to improving the nutritional status of neonatal piglets. Moreover, the use of this caprine product, which intensive farms often have problems disposing of and can potentially pose an environmental issue, would provide an added benefit that should not be underestimated.

## Figures and Tables

**Table 1:** Chemical composition and immunoglobulins of sow and goat colostrum from the first (D1) and the second (D2) day of postpartum milking.

Item	Sow	Goat D1	Goat D2	SEM	*p* Value
Dry matter, %	26.8 ^a^	24.7 ^b^	22.1 ^c^	0.38	<0.001
Crude Protein, %	18.5 ^a^	10.5 ^b^	7.4 ^c^	0.29	<0.001
Crude Fat, %	5.5 ^b^	9.0 ^a^	9.1 ^a^	0.30	<0.001
Lactose, %	2.8 ^b^	3.4 ^a^	3.8 ^a^	0.07	<0.001
IgG, mg/mL	91.2 ^a^	37.5 ^b^	16.7 ^b^	6.27	<0.001

SEM: standard error of the mean. a–c: different superscript letters in the same row indicate significant differences (*p* < 0.05). Sow colostrum *n* = 15; goat D1 *n* = 5 and goat D2 *n* = 5 (goat colostrum from 5 different batches of 50 goats each one).

**Table 2:** Fatty acid profile (g per 100 g of total fatty acid methyl esters) in sow colostrum fat from different parities.

Fatty Acid	Parity 1	Parity 2–4	Parity ≥ 5	SEM	*p*-Value
10:0	0.01	0.01	0.01	0.001	0.277
12:0	0.04	0.03	0.03	0.005	0.558
14:0	1.52	1.49	1.43	0.089	0.898
15:0	0.15	0.14	0.14	0.007	0.792
16:0	21.75	23.06	23.39	0.411	0.276
17:0	0.33	0.32	0.33	0.017	0.993
18.0	5.51	6.02	5.32	0.190	0.334
20:0	0.10	0.11	0.10	0.005	0.473
21:0	0.03	0.02	0.02	0.001	0.297
22:0	0.10	0.12	0.10	0.005	0.469
23:0	0.03 ^a^	0.02 ^b^	0.02 ^b^	0.001	0.017
24:0	0.07	0.08	0.09	0.005	0.301
Total non-branched SFA	29.61	31.41	30.94	0.537	0.421
*iso* 15:0	0.08	0.07	0.07	0.003	0.382
*anteiso* 15:0	0.05	0.05	0.05	0.002	0.992
*iso* 16:0	0.03	0.03	0.03	0.002	0.743
*iso* 17:0	0.05	0.05	0.04	0.003	0.299
*anteiso* 17:0	0.09	0.04	0.08	0.022	0.565
*iso* 18:0	0.16	0.16	0.14	0.011	0.752
Total branched SFA	0.46	0.39	0.42	0.031	0.676
TOTAL SFA	30.10	31.83	31.38	0.552	0.465
*cis*-9 14:1	0.05	0.04	0.04	0.005	0.538
*cis*-7 16:1	1.53	1.58	1.60	0.051	0.829
*cis*-9 16:1	3.93	3.17	3.91	0.220	0.325
*cis*-9 17:1	0.31	0.26	0.28	0.010	0.163
*cis*-9 18:1	36.6	35.6	35.9	0.612	0.810
*cis*-11 18:1	4.96	4.68	5.18	0.132	0.332
*cis*-13 18:1	0.23	0.22	0.25	0.007	0.306
Other *cis*-20:1	0.05	0.05	0.05	0.004	0.769
*cis*-11 20:1	0.25	0.23	0.24	0.017	0.838
*cis*-13 22:1	0.05	0.05	0.05	0.002	0.986
Total *cis* MUFA	47.9	45.8	47.5	0.725	0.509
*trans*-16:1	0.07	0.07	0.06	0.003	0.674
*trans*-18:1	0.46	0.45	0.41	0.022	0.669
Total *trans* MUFA	0.52	0.52	0.47	0.024	0.645
TOTAL MUFA	48.44	46.35	47.93	0.706	0.496
Other *trans*-*trans*	0.04	0.04	0.04	0.004	0.980
*trans*-9 *trans*-12	0.02	0.02	0.02	0.001	0.873
*cis*-9 *trans*-13 + *trans*-8 *cis*-12	0.04	0.03	0.03	0.003	0.249
*trans*-8 *cis*-13 + *cis*-9 *trans*-12	0.09	0.08	0.09	0.005	0.622
*trans*-11 *cis*-15 + *trans*-10 *cis*-15	0.04	0.03	0.04	0.004	0.511
*cis*-9 *cis*-12	16.39	16.61	15.80	0.431	0.724
Total non-conjugated 18:2	16.63	16.80	16.01	0.439	0.731
*cis*-9 *trans*-11	0.06 ^a^	0.06 ^a^	0.05 ^b^	0.002	0.006
*trans*-9 *cis*-11	0.01	0.01	0.01	0.001	0.970
*trans*-11 *trans*-13	0.02	0.02	0.02	0.002	0.501
*trans*-8 *trans*-10 + *trans*-9 *trans*-*trans*-11+ *trans*-10 *cis*-12	0.04	0.03	0.03	0.002	0.080
Total conjugated 18:2	0.14 ^a^	0.12 ^b^	0.11 ^c^	0.003	0.010
TOTAL 18:2	16.76	16.92	16.12	0.440	0.724
18:3 n-6	0.36	0.29	0.29	0.028	0.582
18:3 n-3	0.92	0.93	0.87	0.031	0.678
20:2 n-6	0.40	0.40	0.38	0.013	0.746
20:3 n-6	0.27	0.31	0.28	0.011	0.355
20:3 n-3	0.08	0.08	0.08	0.003	0.597
20:4 n-6	1.13	1.26	1.13	0.046	0.462
22:2 n-6	0.04	0.04	0.04	0.001	0.810
20:5 n-3 EPA	0.08	0.08	0.07	0.005	0.667
22:4 n-6	0.24	0.25	0.21	0.013	0.472
22:5 n-6	0.03	0.04	0.03	0.003	0.440
22:5 n-3 DPA	0.35	0.40	0.34	0.024	0.610
22:6 n-3 DHA	0.12	0.14	0.12	0.008	0.663
Total other PUFA	4.03	4.21	3.84	0.128	0.503
Total omega-3	1.57	1.63	1.48	0.053	0.522
Total omega-6	18.85	19.19	18.16	0.472	0.657
TOTAL PUFA	20.80	21.13	19.96	0.531	0.644
omega-6/omega-3	12.07	11.81	12.33	0.195	0.569

SEM: standard error of the mean; SFA: saturated fatty acids; MUFA: monounsaturated fatty acids; EPA: eicosapentaenoic acid; DPA: docosapentaenoic acid; DHA: docosahexaenoic acid; PUFA: polyunsaturated fatty acids. ^a, b, c^ Means with different superscripts indicate significant differences between samples. Parity 1 *n* = 5, parity 2–4 *n* = 5 and parity ≥ 5 *n* = 5.

**Table 3:** Saturated fatty acid profile (g per 100 g of total fatty acid methyl esters) in goat fat colostrum from the first (D1) and the second (D2) day of postpartum milking.

	Collection Day			
Fatty Acid	D1	D2	SEM	*p*-Value
4:0	2.00	2.66	0.097	0.012
5:0	0.01	0.02	0.001	0.038
6:0	1.72	2.44	0.085	0.004
7:0	0.02	0.02	0.001	0.013
8:0	1.62	2.42	0.082	0.002
9:0	0.02	0.03	0.001	0.005
10:0	4.95	7.02	0.169	0.000
11:0	0.03	0.03	0.002	0.079
12:0	2.67	3.24	0.045	0.000
13:0	0.04	0.04	0.002	0.553
14:0	11.57	11.75	0.112	0.456
15:0	0.51	0.49	0.011	0.531
16:0	31.56	29.11	0.362	0.012
17:0	0.59	0.57	0.007	0.178
18.0	7.77	7.41	0.156	0.292
20:0	0.17	0.15	0.007	0.213
22:0	0.06	0.05	0.002	0.029
23:0	0.01	0.01	0.001	0.173
24:0	0.02	0.01	0.002	0.301
Total non-branched SFA	65.32	67.47	0.275	0.006
*iso* 13:0	0.01	0.01	0.000	0.263
*anteiso* 13:0	0.02	0.02	0.000	0.020
*iso* 14:0	0.03	0.03	0.001	0.909
*iso* 15:0	0.15	0.15	0.004	0.846
*anteiso* 15:0	0.13	0.12	0.003	0.335
*iso* 16:0	0.17	0.16	0.006	0.117
*iso* 17:0	0.43	0.41	0.006	0.113
*anteiso* 17:0	0.35	0.36	0.001	0.586
*iso* 18:0	0.06	0.07	0.020	0.035
Total branched SFA	1.34	1.32	0.020	0.593
TOTAL SFA	66.66	68.79	0.290	0.008

SEM: standard error of the mean; SFA: saturated fatty acids. D1 *n* = 5 and D2 *n* = 5 (goat colostrum from 5 different batches of 50 goats each one).

**Table 4:** Unsaturated fatty acid profile (g per 100 g of total fatty acid methyl esters) in goat fat colostrum from the first (D1) and the second (D2) day of postpartum milking.

	Collection Day			
Fatty Acid	D1	D2	SEM	*p*-Value
10:1	0.10	0.14	0.006	0.005
*cis*-9 12:1	0.004	0.01	0.000	0.490
*cis*-11 12:1	0.03	0.04	0.001	0.010
*cis*-9 14:1	0.13	0.15	0.004	0.202
*cis*-7 16:1	0.44	0.41	0.009	0.066
*cis*-9 16:1	1.06	1.08	0.021	0.598
*cis*-9 17:1	0.33	0.35	0.012	0.615
*cis*-9 18:1	22.00	20.40	0.270	0.021
*cis*-11 18:1	1.08	1.05	0.025	0.479
*cis*-12 18:1	0.25	0.24	0.005	0.345
*cis*-13 18:1	0.11	0.10	0.003	0.137
*cis*-14 18:1	0.09	0.09	0.002	0.246
*cis*-15 18:1	0.05	0.04	0.001	0.028
*cis*-16 18:1	0.03	0.02	0.002	0.056
*cis*-11 20:1	0.09	0.07	0.003	0.068
*cis*-13 22:1	0.03	0.02	0.001	0.019
Total *cis* MUFA	25.83	24.20	0.283	0.023
*trans*-15:1	0.03	0.03	0.001	0.468
Other *trans*-16:1	0.30	0.30	0.007	0.652
*trans*-6+7+8 16:1	0.08	0.08	0.002	0.223
*trans*-9 16:1	0.06	0.05	0.006	0.257
*trans*-4 18:1	0.02	0.02	0.002	0.149
*trans*-5 18:1	0.02	0.02	0.001	0.882
*trans*-6+7+8 18:1	0.19	0.16	0.004	0.011
*trans*-9 18:1	0.23	0.20	0.007	0.047
*trans*-10 18:1	0.28	0.36	0.018	0.067
*trans*-11 18:1	1.36	1.22	0.034	0.064
*trans*-12 18:1	0.31	0.29	0.007	0.312
Total *trans* MUFA	2.88	2.73	0.047	0.152
TOTAL MUFA	28.71	26.92	0.267	0.012
Other *trans*-*trans*	0.10	0.10	0.003	0.313
*cis*-9 *trans*-12 + *trans*-8 *cis*-12	0.14	0.14	0.002	0.102
*trans*-8 *cis*-13 + *cis*-9 *trans*-12	0.08	0.06	0.002	0.029
*trans*-9 *cis*-12	0.01	0.01	0.001	0.053
*trans*-11 *cis*-15 + *trans*-10 *cis*-15	0.02	0.01	0.001	0.013
*cis*-9 *cis*-12	2.47	2.22	0.031	0.005
*cis*-12 *cis*-15	0.05	0.03	0.001	0.001
Total non-conjugated 18:2	2.87	2.56	0.038	0.005
*cis*-9 *trans*-11	0.58	0.51	0.021	0.130
*trans*-9 *cis*-11	0.03	0.02	0.001	0.241
*trans*-11 *cis*-13	0.02	0.02	0.002	0.147
*trans*-8 *trans*-10 + *trans*-9 *trans*-11+ *trans*-10 *trans*-12	0.02	0.02	0.001	0.178
Total conjugated 18:2	0.65	0.57	0.023	0.123
TOTAL 18:2	3.52	3.13	0.056	0.010
18:3 n-6	0.03	0.03	0.001	0.169
18:3 n-3	0.10	0.10	0.003	0.604
20:2 n-6	0.03	0.03	0.002	0.409
*cis*-9 *trans*-11 *cis*-15 18:3	0.04	0.04	0.004	0.582
20:3 n-6	0.04	0.03	0.003	0.226
20:4 n-6	0.40	0.40	0.014	0.458
20:5 n-3 EPA	0.03	0.03	0.001	0.558
22:4 n-6	0.10	0.10	0.004	0.747
22:5 n-6	0.02	0.02	0.001	0.153
22:5 n-3 DPA	0.11	0.10	0.005	0.310
22:6 n-3 DHA	0.02	0.02	0.002	0.618
TOTAL Other PUFA	0.90	0.86	0.032	0.573
Total omega-3	0.30	0.28	0.012	0.538
Total omega-6	3.07	2.80	0.050	0.029
TOTAL PUFA	4.43	4.00	0.081	0.033
omega-6/omega-3	10.45	9.95	0.284	0.409

MUFA: monounsaturated fatty acids; EPA: eicosapentaenoic acid; DPA: docosapentaenoic acid; DHA: docosahexaenoic acid; PUFA: polyunsaturated fatty acids. D1 *n* = 5 and D2 *n* = 5 (goat colostrum from 5 different batches of 50 goats each one).

## Data Availability

The data presented in this study are available on request from the corresponding author (P.G.-C.).
